# Hypercoagulability progresses to hypocoagulability during evolution of acetaminophen-induced acute liver injury in pigs

**DOI:** 10.1038/s41598-017-09508-3

**Published:** 2017-08-24

**Authors:** Karla Chui Luan Lee, Luisa Baker, Susan Mallett, Anne Riddell, Pratima Chowdary, Hatim Alibhai, Yu-Mei Chang, Simon Priestnall, Giacomo Stanzani, Nathan Davies, Rajeshwar Mookerjee, Rajiv Jalan, Banwari Agarwal

**Affiliations:** 1 Department of Clinical Science and Services, The Royal Veterinary College, University of London, Hertfordshire, UK; 2 Liver Failure Group, Institute of Liver and Digestive Health, University College London Medical School Royal Free Campus, London, UK; 3 Department of Anaesthesia, Royal Free Hospital, Royal Free London NHS Foundation Trust, London, UK; 4 Katherine Dormandy Haemophilia and Thrombosis Centre, Royal Free Hospital, Royal Free London NHS Foundation Trust, London, UK; 5 Department of Research Support, The Royal Veterinary College, University of London, Hertfordshire, UK; 6 Department of Pathobiology and Population Sciences, The Royal Veterinary College, University of London, Hertfordshire, UK; 7 Department of Intensive Care Medicine, Royal Free Hospital, Royal Free London NHS Foundation Trust, London, UK

## Abstract

Increases in prothrombin time (PT) and international normalised ratio (INR) characterise acute liver injury (ALI) and failure (ALF), yet a wide heterogeneity in clotting abnormalities exists. This study defines evolution of coagulopathy in 10 pigs with acetaminophen (APAP)-induced ALI compared to 3 Controls. APAP administration began at 0 h and continued to ‘ALF’, defined as INR >3. In APAP pigs, INR was 1.05 ± 0.02 at 0 h, 2.15 ± 0.43 at 16 h and > 3 at 18 ± 1 h. At 12 h thromboelastography (TEG) demonstrated increased clot formation rate, associated with portal vein platelet aggregates and reductions in protein C, protein S, antithrombin and A Disintegrin and Metalloprotease with Thrombospondin type 1 repeats–13 (ADAMTS-13) to 60%, 24%, 47% and 32% normal respectively. At 18 ± 1 h, INR > 3 was associated with: hypocoagulable TEG profile with heparin-like effect; falls in thrombin generation, Factor V and Factor VIII to 52%, 19% and 17% normal respectively; further decline in anticoagulants; thrombocytopenia; neutrophilia and endotoxemia. Multivariate analysis, found that ADAMTS-13 was an independent predictor of a hypercoagulable TEG profile and platelet count, endotoxin, Protein C and fibrinogen were independent predictors of a hypocoagulable TEG profile. INR remained normal in Controls. Dynamic changes in coagulation occur with progression of ALI: a pro-thrombotic state progresses to hypocoagulability.

## Introduction

Acute liver injury (ALI) is characterised by coagulopathy, as assessed by elevation in Prothrombin Time (PT) and its derivative International Normalised Ratio (INR). Severe ALI, when complicated by hepatic encephalopathy is termed acute liver failure (ALF)^1, 2^. Traditionally, it has been believed that elevations in PT and INR in ALI/ALF patients are associated with a bleeding diathesis. However, a number of reports over the past 10 years have failed to demonstrate clinically significant bleeding (spontaneous or intervention related), some even suggesting an increased tendency to clot formation^3–6^. This observation supports the concept of dysregulated haemostasis with an increased thrombotic tendency and decreased haemostatic reserve with potential decreased bleeding tendency that was described initially in chronic liver disease^7^, but also observed in ALI and ALF^8^. The mechanisms for this phenomenon seem multifactorial: proportional decline in the procoagulants and the natural anticoagulants produced by the liver; enhanced endothelial production and activation of Factor (F) VIII and von Willebrand Factor (vWF); release of procoagulant microparticles from monocytes, platelets and endothelium; and dampened fibrinolytic capacity^5, 6, 8–11^.

The studies of ALI/ALF patients indicate a wide heterogeneity in clotting abnormalities. In a study of 20 ALF patients, none had bleeding complications, but two had frequent thrombosis of continuous renal replacement therapy (CRRT) filters^5^. This is consistent with previous reports of short CRRT filter life in ALF patients^12^. In a study of 51 patients with ALF or ALI, nine had bleeding complications, but thrombosis was reported in nine including bowel ischaemia, limb ischaemia, portal vein thrombosis, and CRRT catheter thrombosis^6^. It is possible that the large heterogeneity in coagulation disturbances observed in ALI/ALF patients is due in part to the cross-sectional nature of study designs, resulting in inclusion of patients at the time of admission to the Intensive Care Unit, rather than at specific stages of liver failure or at the onset of specific complications of liver failure. Complications such as superimposed infection; endothelial dysfunction; and renal failure requiring extracorporeal therapies and use of inotropes may all independently contribute to coagulation disturbances. Bacterial endotoxin is known to activate coagulation and fibrinolysis, initiated by cytokine-induced release of tissue factor (TF) from circulating monocytes^13–16^. Hepatic vascular endothelial injury in ALI leads to the release of endogenous heparinoids, e.g. heparan sulphate from the liver, leading to a heparin-like effect^17, 18^. Development of acute kidney injury in ALI/ALF results in significant changes in platelet counts and concentrations of procoagulants and anticoagulants^19^. Epinephrine infusion results in dose dependent increase in FVIII clotting activity, vWF antigen (vWF Ag), tissue type plasminogen activator and platelets^20^. Therefore, at present, the evolution of coagulation disturbance in the various stages of ALI progression and the associated mechanisms are unknown.

We hypothesised that progression of coagulation disturbances would be associated with endotoxemia and systemic inflammation and that coagulation changes in the systemic circulation may not reflect changes in the portal circulation due to gut derived endotoxin and portal vein blood flow. The aim of the current study was therefore to investigate the evolution of coagulopathy in a well-described paracetamol (acetaminophen, APAP)-induced pig model of ALF prior to onset of multi-organ failure and without bacterial infection. Coagulopathy and underlying mechanisms for coagulation disturbance were assessed longitudinally in systemic and portal blood from the time of induction of liver injury up to the development of full-blown liver failure.

## Results

### Acetaminophen (APAP) treatment leads to acute liver injury (ALI) and failure (ALF) associated with elevation in International Normalised Ratio (INR)

**Figure 1:**
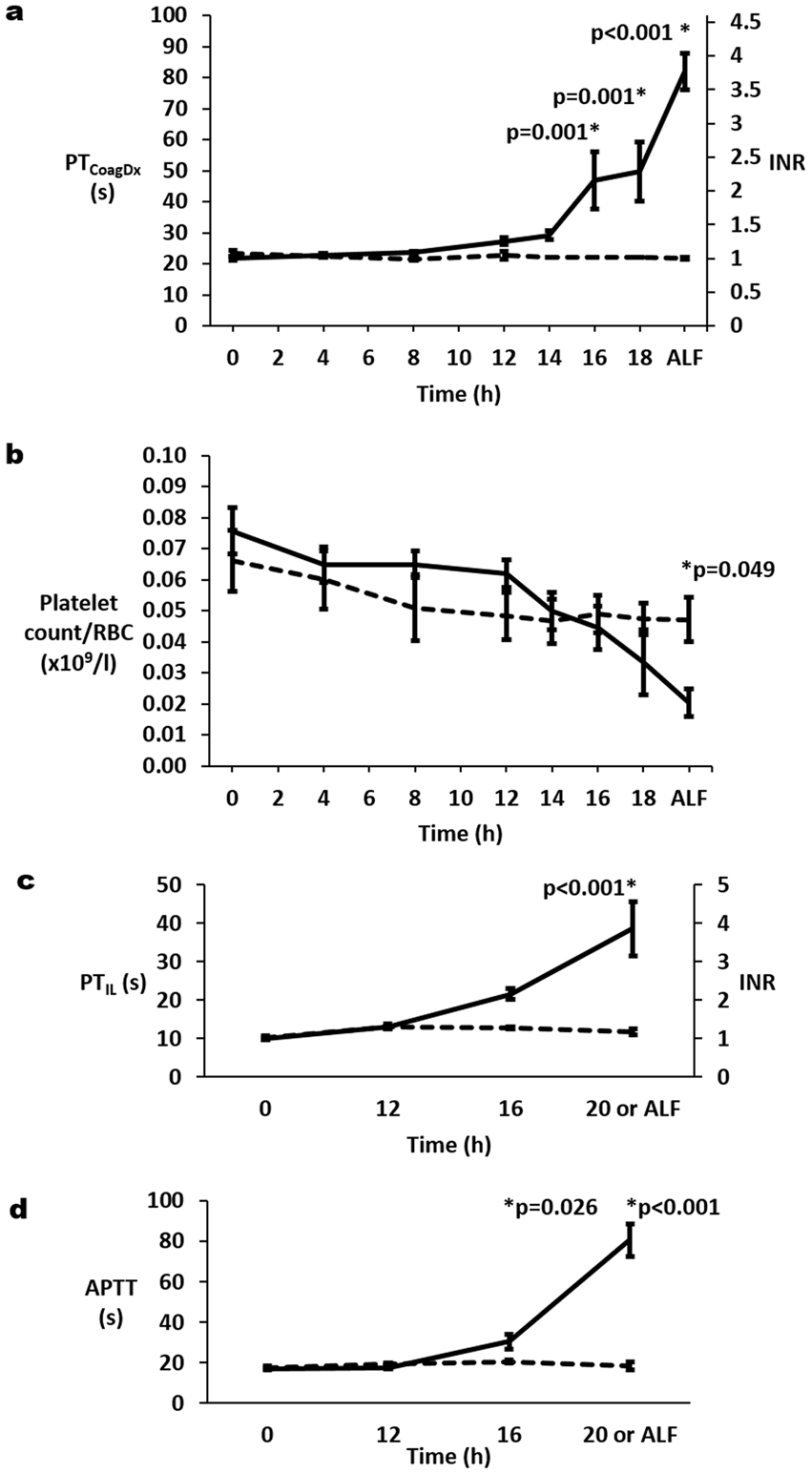
Change in (**a**) bedside PT_CoagDx_ and (**b**) platelet count (expressed as a ratio of red blood cell count (platelet count/RBC)) with progression through acute liver injury to acute liver failure (ALF), which was defined as international normalised ratio (INR) of 3 or more and change in (**c**) prothrombin time (PT_IL_) and (**d**) activated partial thromboplastin time (APTT) in stored samples at 0 h, 12 h, 16 h and ALF. Mean ( ± se) for each parameter is plotted for acetaminophen (APAP) treated pigs (solid lines) and control pigs (broken lines). P values are given above significant differences between the APAP and Control groups.

APAP treatment in 10 pigs resulted in non-significant increases in INR (calculated from a bedside prothrombin time (PT_CoagDx_) analyser) from 1.05 ± 0.02 at 0 h to 1.26 ± 0.05 at 12 h (p = 0.265) and to 1.34 ± 0.07 at 14 h (p = 0.137). However by 16 h, a significant increase in INR to 2.15 ± 0.43 (p < 0.001) was seen and at 18 ± 1 h INR exceeded 3 (p < 0.001), indicative of ‘ALF’ (Fig. 1). Total dose of APAP administered to achieve ‘ALF’ was 46.0 ± 2.9 g. In three Control pigs, placebo treatment resulted in no change in INR from 0 h to 20 h.

In the APAP group, APAP-induced hepatotoxicity at the time INR exceeded 3 was further confirmed by demonstrating significant elevation in total serum high-mobility group box-1 protein (HMGB1)^21^. In the APAP group total HMGB1 was 1.4 ± 0.1 ng/ml and 5.0 ± 1.0 ng/ml at 0 h and ‘ALF’ respectively (p = 0.003). Whereas, in the Control group total HMGB1 was 1.5 ± 0.1 ng/ml and 1.2 ± 0.1 ng/ml at 0 h and 20 h respectively. In addition, liver histopathology at death or sacrifice, which occurred within 20 h of ‘ALF’, revealed acute centrilobular and midzonal hepatocyte degeneration and necrosis in all APAP pigs.

### Progression of ALI in APAP pigs is associated with significant neutrophilia and thrombocytopenia, but no evidence of blood loss

Red blood cell count (RBC) decreased significantly in the Controls from 5.3 ± 0.1 × 10^12^/l at 0 h to 4.3 ± 0.1 × 10^12^/l (p < 0.001) at 20 h. This was likely due to hemodilution by intravenous fluid therapy dictated by the study protocols. Conversely RBC increased significantly in the APAP group from 5.7 ± 0.1 × 10^12^/l at 0 h to 5.9 ± 0.1 × 10^12^/l at ‘ALF’ (p < 0.001), likely due to hemoconcentration as a result of capillary leak with progression towards ‘ALF’. Platelet count, confirmed by blood smears, declined significantly (p < 0.001) in both Controls (from 350 ± 44 × 10^9^/l at 0 h to 205 ± 33 × 10^9^/l at 20 h) and the APAP group (from 417 ± 39 × 10^9^/l at 0 h to 125 ± 29 × 10^9^/l at ‘ALF’). To account for differential hemoconcentration/hemodilution between groups, platelet count was expressed as platelets per red blood cell (platelet count/RBC) for group comparisons (Fig. 1). For Controls, platelet count/RBC decreased significantly from 0 h to 12 h (p = 0.001), but remained stable thereafter. For the APAP group, platelet count/RBC decreased significantly from 0 h to 12 h (p = 0.001) and continued to decrease thereafter to ‘ALF’ (p < 0.001), resulting in a lower platelet count/RBC in the APAP group compared to Controls (p = 0.049) suggesting potential consumption of platelets in the APAP group. APAP pigs demonstrated significant progressive increases in total white blood cells (from 14.5 ± 2.1 × 10^9^/l at 0 h to 28.6 ± 3.2 × 10^9^/l at ‘ALF’, p < 0.001), neutrophils (from 7.6 ± 1.6 × 10^9^/l at 0 h to 21.2 ± 2.9 × 10^9^/l at ‘ALF’, p < 0.001) and band neutrophils (from 0.0 ± 0.0 × 10^9^/l at 0 h to 1.4 ± 0.5 × 10^9^/l at ‘ALF’, p < 0.001). Significant changes in white blood cell counts were not seen in Control pigs.

No bleeding complications were observed in any animal. However, the portal vein catheters thrombosed in 4 out of 10 APAP pigs by 12 h in 3 pigs and by 14 h in 1 pig.

### Progression of ALI is associated first with a hypercoagulable thromboelastography (TEG) profile followed by a hypocoagulable TEG profile with a heparin-like effect

**Figure 2:**
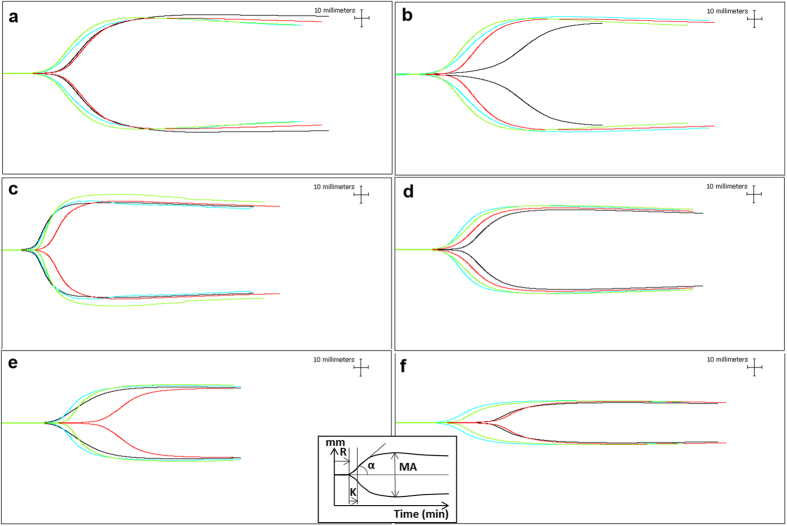
Change in thromboelastography (TEG) profiles with progression of acute liver injury to acute liver failure (ALF). Representative TEG traces for (**a**) normal pig blood and blood taken from an acetaminophen (APAP) treated pig at times related to onset of APAP dosing: (**b**) 0 h; (**c**) 12 h; (**d**) 16 h; (**e**) 18 h and (**f**) at ‘acute liver failure’. Insert demonstrates TEG parameters: R, reaction time; K, time from beginning of clot formation until amplitude reaches 20 mm; α, angle; and MA, maximum amplitude. Traces are colour coded: black, femoral arterial blood in natural cup; blue, femoral arterial blood in heparinase cup; red, portal vein blood in natural cup; and green, portal vein blood in heparinase cup.

Thromboelastography was used to monitor global changes in coagulation in portal vein and arterial blood samples with progression of ALI. Results are given in Tables 1 and 2. Representative traces for APAP pigs are shown in Fig. 2. Overall TEG results were significantly affected by time (p < 0.001) and the use of heparinase (p < 0.001) for both arterial and portal vein blood. However, there was no significant difference in the TEG results from portal vein samples compared to arterial samples.

**Table 1:** Thromboelastography (TEG) results from acetaminophen (APAP) treated pigs with progression of acute liver injury to acute liver failure (ALF).

	Time	Femoral artery			Portal vein		
		Natural cup	Heparinase cup		Natural cup	Heparinase cup	
**R (min)**	**Normal**	**18.2 ± 1.6, n = 9**	**13.5 ± 1.1, n = 9**				
	**0 h**	20.1 ± 1.9, n = 9	13.0 ± 0.7, n = 9		19.9 ± 1.6, n = 10	16.7 ± 1.6, n = 10	
	**12 h**	15.7 ± 1.4, n = 8	11.7 ± 0.9, n = 8		18.1 ± 4.0, n = 6	13.8 ± 2.5, n = 7	
	**14 h**	23.1 ± 1.9, n = 9	12.7 ± 1.1, n = 9		24.1 ± 4.3, n = 6	15.0 ± 1.8, n = 7	
	**16 h**	25.0 ± 0.9, n = 7	14.7 ± 0.8, n = 7		24.4 ± 4.0, n = 5	17.0 ± 0.8, n = 5	
	**18 h**	26.4 ± 5.6, n = 3	16.8 ± 1.5, n = 4		38.1 ± 5.7, n = 2	21.6 ± 0.6, n = 4	
	**‘ALF’**	46.5 ± 17.4, n = 9, p < 0.001	17.0 ± 1.1, n = 9	p < 0.001	46.1 ± 12.5, n = 6, p < 0.001	21.8 ± 1.2, n = 6	p = 0.002
**K (min)**	**Normal**	**9.2 ± 1.9, n = 9**	**7.2 ± 1.0, n = 9**				
	**0 h**	15.8 ± 3.3, n = 9, p = 0.002	8.5 ± 0.9, n = 9	p = 0.001	13.2 ± 3.6, n = 10	8.7 ± 1.3, n = 10	p = 0.026
	**12 h**	4.8 ± 0.3, n = 8, p = 0.036	4.6 ± 0.4, n = 8		6.1 ± 1.3, n = 6, p = 0.002	5.3 ± 1.6, n = 7	
	**14 h**	9.5 ± 1.8, n = 9	5.2 ± 0.5, n = 9	p = 0.040	9.6 ± 2.4, n = 6	4.0 ± 0.5, n = 7, p = 0.027	p = 0.036
	**16 h**	8.1 ± 1.6, n = 7	4.9 ± 0.3, n = 7		8.2 ± 1.6, n = 5, p = 0.025	4.9 ± 0.7, n = 5	
	**18 h**	8.9 ± 0.7, n = 3	6.1 ± 0.4, n = 4		12.2 ± 2.6, n = 2	6.0 ± 0.4, n = 4	
	**‘ALF’**	13.7 ± 1.5, n = 8, p = 0.050	10.6 ± 1.2, n = 9		14.3 ± 1.4, n = 5	10.8 ± 1.6, n = 6	

Parameters listed include: R, time to initial fibrin formation and K, speed of clot formation. Times listed are: Normal, prior to any intervention; hours from onset of APAP dosing; and ‘ALF’, time at which international normalised ratio exceeded 3. n represents sample number. p values given in same cell as data represent significant differences compared to Normal. p values given in column to the right of data represent significant differences between adjacent natural and heparinase cups.

**Table 2:** Thromboelastography (TEG) results from acetaminophen (APAP) treated pigs with progression of acute liver injury to acute liver failure (ALF).

	Time	Femoral artery			Portal vein		
		Natural cup	Heparinase cup		Natural cup	Heparinase cup	
**α** (°)	**Normal**	**30.8 ± 4.8, n = 9**	**32.6 ± 4.1, n = 9**				
	**0 h**	20.5 ± 3.3, n = 9, p = 0.010	26.7 ± 2.4, n = 9		27.3 ± 3.9, n = 10	28.6 ± 3.2, n = 10	
	**12 h**	40.1 ± 1.3, n = 8, p = 0.021	41.7 ± 1.7, n = 8, p = 0.025		37.3 ± 5.1, n = 6	41.2 ± 5.7, n = 7, p = 0.026	
	**14 h**	27.1 ± 3.8, n = 9	38.0 ± 2.8, n = 9	p = 0.008	29.9 ± 6.8, n = 6	46.1 ± 3.4, n = 7, p = 0.001	p = 0.001
	**16 h**	26.6 ± 2.2, n = 7	37.9 ± 1.5, n = 7	p = 0.014	26.8 ± 2.6, n = 5	38.9 ± 3.3, n = 5	p = 0.026
	**18 h**	22.2 ± 1.4, n = 3	31.4 ± 1.4, n = 4		16.6 ± 3.6, n = 2, p = 0.046	30.0 ± 1.1, n = 4	p = 0.041
	**‘ALF’**	14.1 ± 2.0, n = 9, p < 0.001	20.8 ± 1.8, n = 9, p = 0.005		12.0 ± 2.5, n = 6, p < 0.001	20.4 ± 2.4, n = 6, p = 0.014	
**MA (mm)**	**Normal**	**68.6 ± 2.0, n = 9**	**68.4 ± 1.5, n = 9**				
	**0 h**	62.8 ± 2.5, n = 9, p = 0.033	68.9 ± 1.3, n = 9		65.4 ± 2.4, n = 10	65.9 ± 1.5, n = 10	
	**12 h**	61.2 ± 1.6, n = 8, p = 0.011	62.9 ± 1.1, n = 8		61.4 ± 1.3, n = 6, p = 0.029	59.9 ± 1.6, n = 7, p = 0.006	
	**14 h**	54.4 ± 1.8, n = 9, p < 0.001	57.6 ± 1.4, n = 9, p < 0.001		53.3 ± 2.5, n = 6, p < 0.001	57.9 ± 1.8, n = 7, p < 0.001	
	**16 h**	47.5 ± 2.1, n = 5, p < 0.001	51.0 ± 3.0, n = 7, p < 0.001		47.4 ± 2.1, n = 5, p < 0.001	52.7 ± 2.3, n = 5, p < 0.001	
	**18 h**	40.0 ± 3.1, n = 3, p < 0.001	44.9 ± 2.1, n = 4, p < 0.001		37.6 ± 1.9, n = 2, p < 0.001	43.0 ± 2.1, n = 4, p < 0.001	
	**‘ALF’**	25.2 ± 3.3, n = 9, p < 0.001	28.8 ± 1.7, n = 9, p < 0.001		23.5 ± 4.4, n = 6, p < 0.001	29.3 ± 2.6, n = 6, p < 0.001	

Parameters listed include: α, rapidity of fibrin build-up and cross-linking; and MA, ultimate strength of the fibrin clot. Times listed are: Normal, prior to any intervention; hours from onset of APAP dosing; and ‘ALF’, time at which international normalised ratio exceeded 3. n represents sample number. p values given in same cell as data represent significant differences compared to Normal. p values given in column to the right of data represent significant differences between adjacent natural and heparinase cups.

At 0 h TEG profiles without heparinase demonstrated significant differences from normal suggestive of hypocoagulability, but these differences were abrogated with heparinase (Tables 1 and 2). As time 0 h was prior to APAP dosing and the observed changes were no longer apparent by 12 h, the TEG profiles at 0 h probably represented contamination with exogenous heparin used during catheter placement.

At 12 h hypercoagulability was evidenced by significant reduction in time required for clot formation (K) in both arterial and portal blood and an increase in rapidity of fibrin build-up and cross-linking (α) in arterial blood. This was however, a transient state with progression to hypocoagulability being demonstrated as early as 12 h on analysis of ultimate strength of the fibrin clot (MA), which showed a significant steady decline from baseline to ‘ALF’ in both arterial and portal blood. Significant increase in time to initial fibrin formation (R) and reduction in α were only seen once INR exceeded 3 at ‘ALF’ in both arterial and portal blood. Addition of heparinase affected TEG results significantly in both arterial and portal blood at 14 h and at the time of ‘ALF’. At 14 h mean K was 45% and 58% lower in the presence of heparinase for arterial and portal blood respectively and at ‘ALF’ mean R was 64% and 53% lower in the presence of heparinase for arterial and portal blood respectively. These results suggest an endogenous heparin-like effect component to hypocoagulability.

### Progression of ALI is associated with dynamic changes in clotting times, thrombin generation, procoagulants (Factor V, Factor VIII, von Willebrand Factor) and anticoagulants (A Disintegrin and Metalloprotease with Thrombospondin type 1 repeats–13, Protein C, Protein S and Antithrombin)

Femoral arterial blood samples were collected and stored at 0 h, 12 h, 16 h and ‘ALF’ from 3 Controls and 8 APAP pigs. Additionally, portal vein blood samples were collected and stored from 2 Controls and 3 APAP pigs at the same times, except that portal vein blood was collected at 14 h instead of 16 h. Prothrombin time (PT_IL_) and activated partial thromboplastin time (APTT) increased with progression of ALI in stored samples from APAP animals (p < 0.001), but not from Controls (Fig. 1). Changes in INR calculated from stored samples mirrored those calculated immediately from a bedside analyser. From 0 h to ‘ALF’, there was a 3.9-fold increase in PT_IL_ (p < 0.001) and 4.7-fold increase in APTT in arterial blood (p < 0.001).

**Figure 3:**
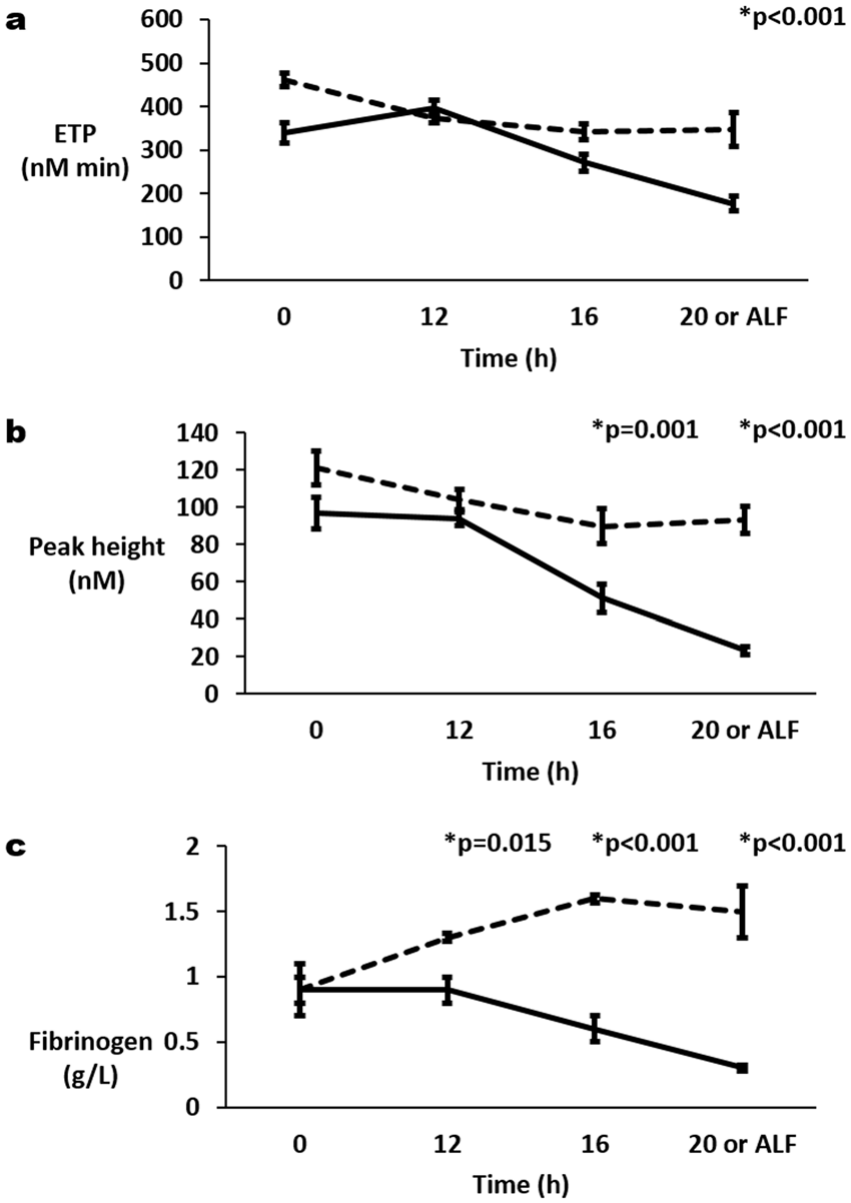
Change in (**a**) endogenous thrombin potential (ETP) and (**b**) peak height for thrombin generation and (**c**) concentrations of fibrinogen in arterial plasma with progression of acute liver injury to acute liver failure (ALF), which was defined as international normalised ratio (INR) of 3 or more. Mean ( ± se) for each parameter are plotted for acetaminophen (APAP) treated pigs (solid lines) and control pigs (broken lines). P values are given above significant differences between the APAP and control groups.

Endogenous thrombin potential (ETP) in arterial and portal blood decreased significantly from 0 h to ‘ALF’ in both the Control and APAP groups, but the decline in the APAP group was greater, resulting in significantly lower ETP in the APAP group compared to Controls at ‘ALF’ (p < 0.001, Fig. 3). At 0 h, ETP in arterial and portal blood from the Control group was 461 ± 16 nM min and 400 ± 56 nM min respectively and from the APAP group was 339 ± 23 nM min and 409 ± 21 nM min respectively. Whereas at ‘ALF’, ETP in arterial and portal blood from the Control group was 347 ± 39 nM min and 340 ± 73 nM min respectively and from the APAP group was 177 ± 17 nM min and 137 ± 11 nM min respectively. Peak Height also decreased significantly in arterial and portal blood with progression to ‘ALF ‘resulting in significant differences between Control and APAP groups at 16 h and ALF (p ≤ 0.001, Fig. 3). At 0 h, Peak Height in arterial and portal blood from the Control group was 121 ± 9 nM and 108 ± 1 nM respectively and from the APAP group was 97 ± 8 nM and 106 ± 2 nM respectively. Whereas at ‘ALF’, Peak Height in arterial and portal blood from the Control group was 93 ± 7 nM and 91 ± 17 nM respectively and from the APAP group was 23 ± 2 nM and 17 ± 1 nM respectively.

**Figure 4:**
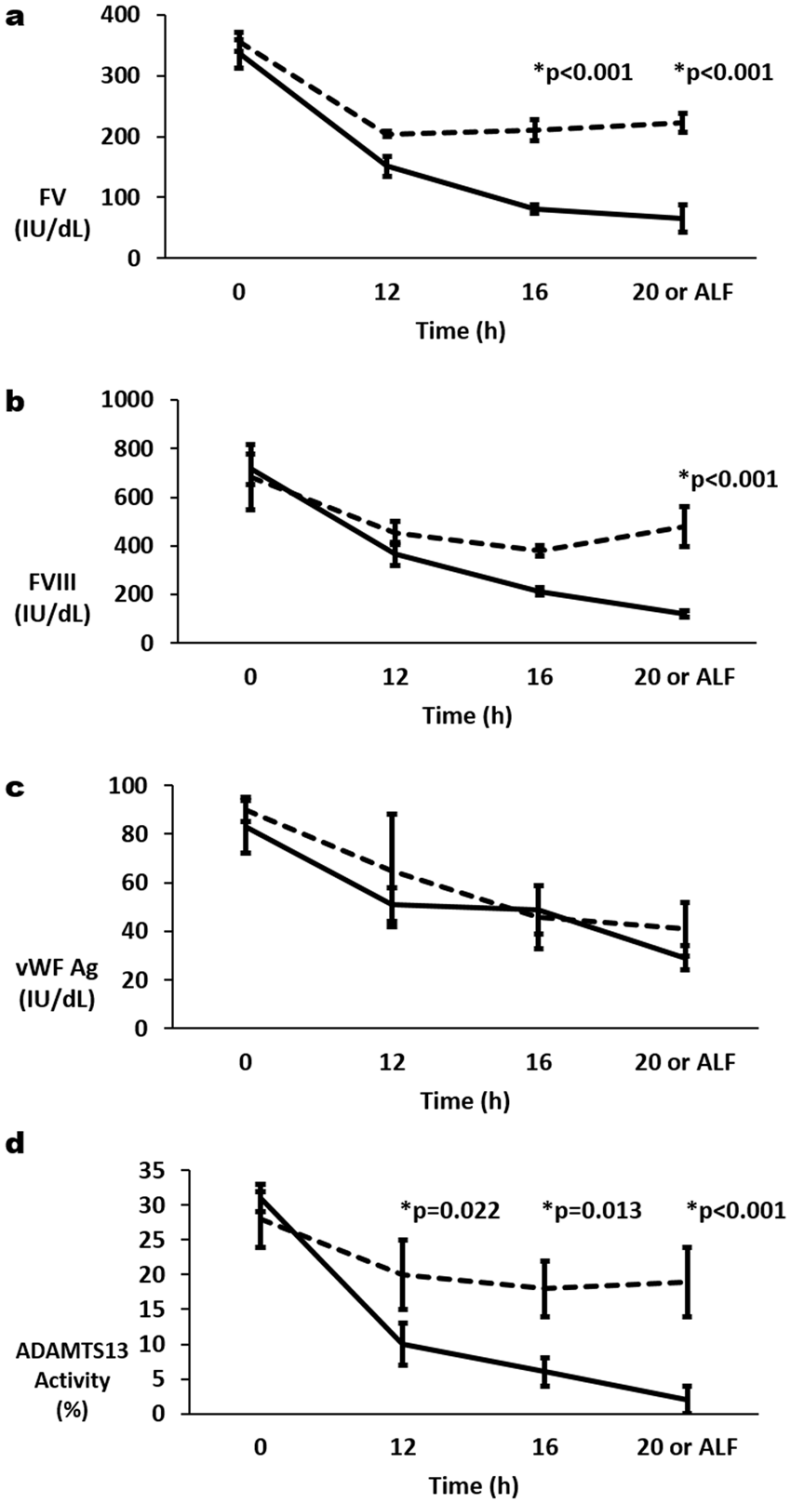
Change in arterial plasma activities/concentrations of (**a**) factor V (FV), (**b**) factor VIII (FVIII), (**c**) von Willebrand factor (vWF Ag) and (**d**) A disintegrin and metalloprotease with thrombospondin type 1 repeats–13 (ADAMTS-13) with progression of acute liver injury to acute liver failure (ALF) which was defined as international normalised ratio (INR) of 3 or more. Mean ( ± se) for each parameter are plotted for acetaminophen (APAP) treated pigs (solid lines) and control pigs (broken lines). P values are given above significant differences between the APAP and control groups.

A significant decrease in mean Factor V (FV) activities was seen between time 0 h and 12 h in Controls and APAP animals with no significant difference between groups (Fig. 4). Decrease in Controls was likely due to aforementioned hemodilution. Mean activities at 12 h compared to 0 h were 47% (p < 0.001), 45% (p < 0.001), 59% (p = 0.002) and 38% (p = 0.001) in arterial Controls, arterial APAP samples, portal vein Controls and portal vein APAP samples respectively. However, after 12 h, FV activities continued to decrease in APAP samples, but not in Controls, leading to significantly lower arterial FV activities at 16 h (p < 0.001) and ‘ALF’ (p < 0.001) in APAP animals compared to Controls. Mean activities at 16 h and 20 h compared to 0 h were 59% and 63% respectively in arterial Controls and 24% and 19% respectively in arterial APAP samples. A similar change in Factor VIII (FVIII) activities was seen (Fig. 3), leading to significantly lower (p < 0.001) arterial FVIII activities at ‘ALF’ in APAP animals (mean FVIII activity of 17% compared to 0 h) compared to Controls (mean FVIII activity of 70% compared to 0 h).

Overall there was a significant effect of time on arterial and portal vein vWF Ag concentrations (p < 0.001) evidenced as decline primarily between 0 h and 12 h (Fig. 4). However, vWF Ag concentration did not differ significantly between the APAP group and Controls at any time. For A Disintegrin and Metalloprotease with Thrombospondin type 1 repeats–13 (ADAMTS-13) activity there was also a significant decline between 0 and 12 h in the APAP group and Controls (Fig. 4). However, the decline in the arterial APAP samples was significantly greater than in the arterial Control samples at 12 h: in APAP and Control samples mean ADAMTS-13 activities at 12 h were 32% and 71% that at 0 h respectively (p = 0.022). This decline continued in the arterial APAP samples to mean activities at ‘ALF’ of 6% that at 0 h.

**Figure 5:**
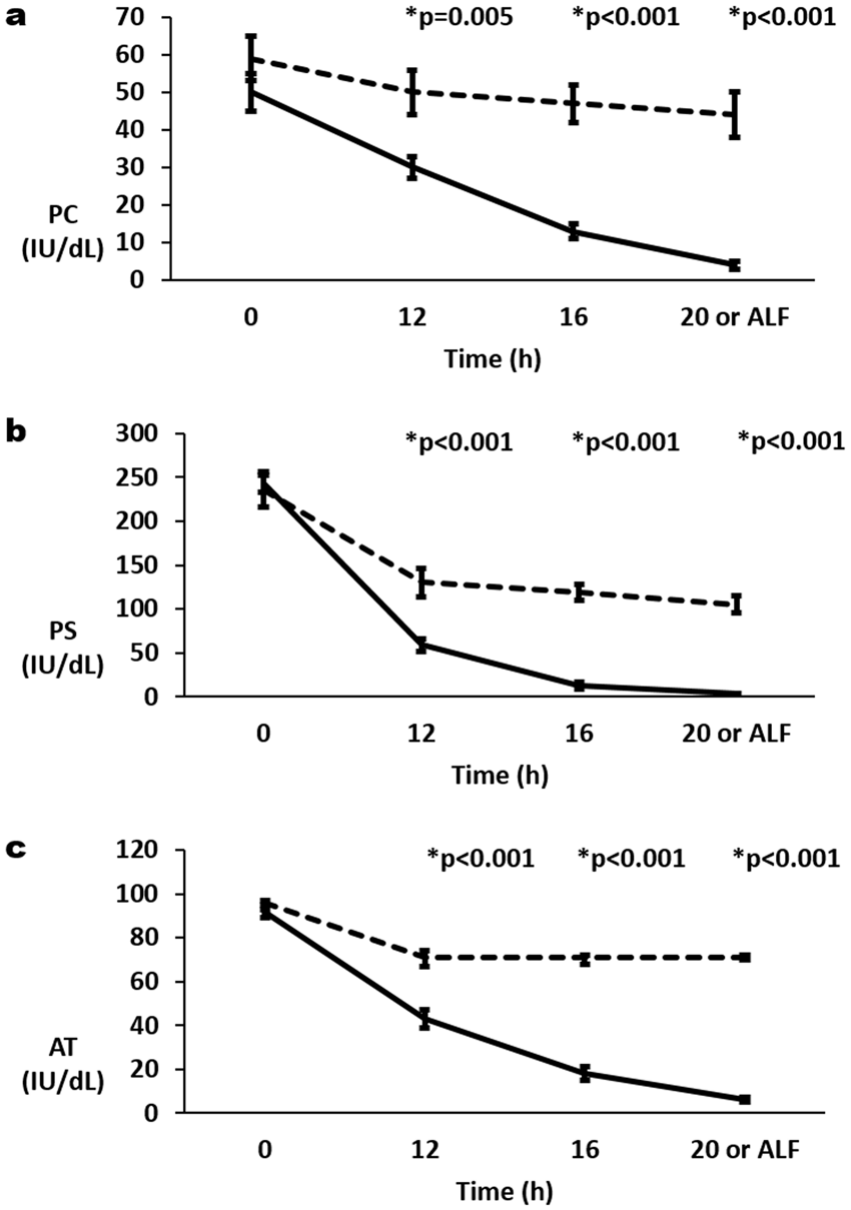
Change in arterial plasma activities of (**a**) protein C (PC), (**b**) protein S (PS) and (**c**) antithrombin (AT) with progression of acute liver injury to acute liver failure (ALF) which was defined as international normalised ratio (INR) of 3 or more. Mean ( ± se) for each parameter are plotted for acetaminophen (APAP) treated pigs (solid lines) and control pigs (broken lines). P values are given above significant differences between the APAP and control groups.

Activities of the natural anticoagulants, Protein C (PC), Protein S (PS) and Antithrombin (AT) all demonstrated similar patterns of decline in arterial and portal vein samples (Fig. 5). Whilst there was a decline in all 3 activities in Control and APAP samples between 0 h and 12 h, the decline in the APAP samples was significantly greater at 12 h (p < 0.005). At 12 h mean arterial Control activities of PC, PS and AT were 85%, 55% and 74% that at 0 h respectively. Whereas at 12 h mean arterial APAP activities of PC, PS and AT were 60%, 24% and 47% that at 0 h respectively. Further decrease in arterial Controls was not seen. In arterial APAP samples, these factors continued to decrease: at ‘ALF’ mean arterial activities of PC, PS and AT were 8%, 1% and 7% that at 0 h respectively (Fig. 5).

Low levels of anti-Xa activity were only detected at time 0 h in two arterial APAP samples (0.07 and 0.24 units/ml) and one arterial Control sample (0.19 units/ml), consistent with TEG results and probably a reflection of exogenous heparin at this time point.

D-dimer concentrations in arterial and portal vein samples did not differ significantly between Controls and the APAP group. Mean fibrinogen concentrations were significantly lower in arterial APAP samples at 12 h compared to Controls (p = 0.015) and all times thereafter to ‘ALF’ (p < 0.001, Fig. 3).

### Portal vein platelet aggregates are observed during hypercoagulable phase of ALI

**Figure 6:**
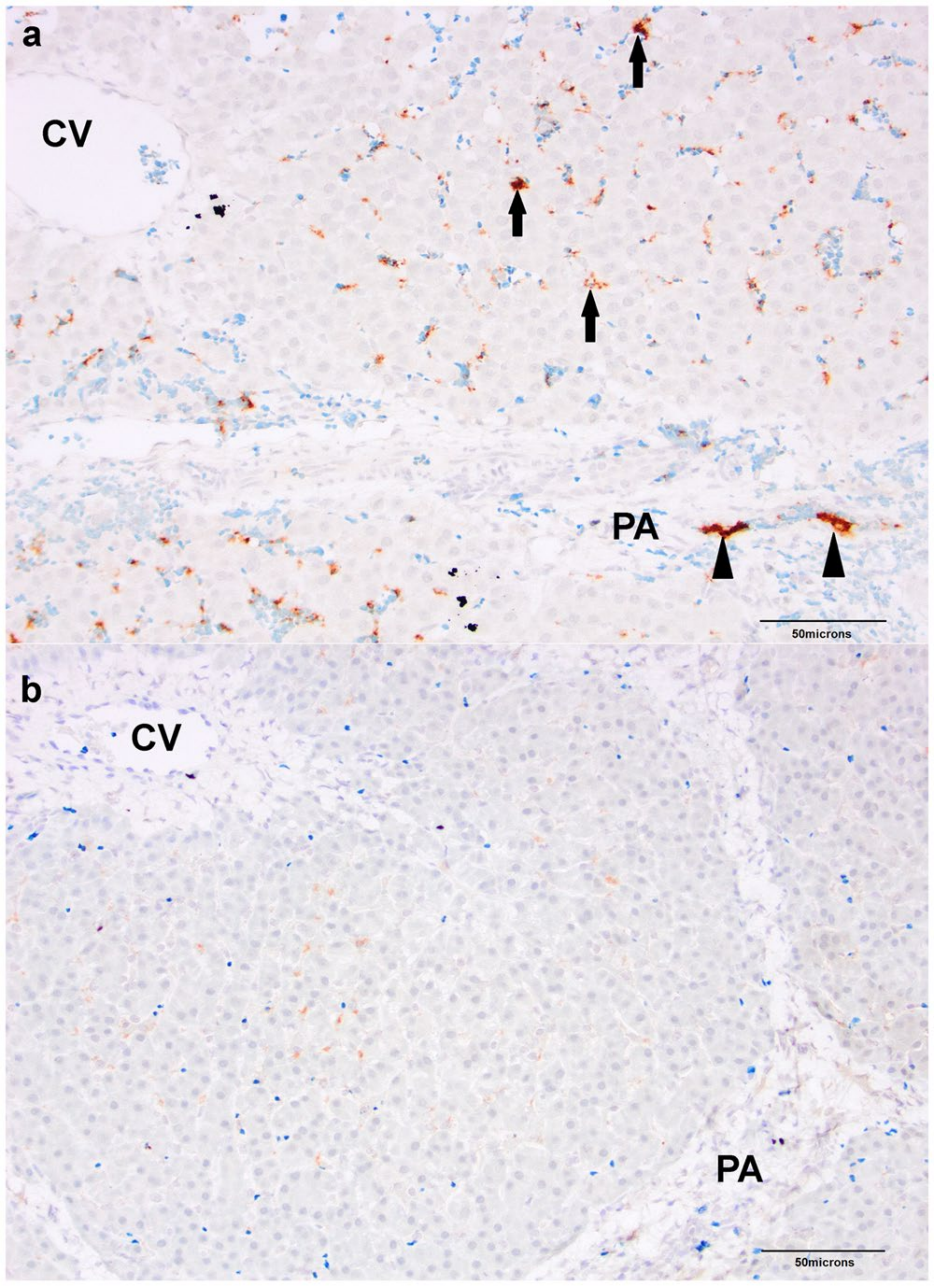
Immunohistochemical lablelling of platelets with CD61 antibody, in liver sections, 12 h after onset of acetaminophen (APAP) dosing when thromboelastography demonstrated a hypercoagulable profile. Portal vein platelet aggregates (arrow heads) were seen in greater numbers in (**a**) APAP treated pigs compared to (**b**) Controls. Sinusoidal platelet aggregates (complete arrows) were not significantly different between groups. PA, portal area; CV, central vein; ×200 magnification.

At 0 h, there was no significant difference in mean number of portal vein platelet aggregates detected per ×10 microscope field in APAP animals (0.81 ± 0.18 per field) compared to Controls (1.14 ± 0.18 per field). However, at 12 h a significant difference was detected with a greater number of portal vein platelet aggregates in the APAP group (1.3 ± 0.5 per field) compared to Controls (0.3 ± 0.2 per field) (Fig. 6). Platelet aggregates were also detected in hepatic sinusoids, but there was no significant effect of time, zone of the hepatic lobule or experimental group on abundance of sinusoidal platelet aggregates.

### Plasma endotoxin increases with progression of ALI

In APAP pigs, plasma endotoxin concentration at ‘ALF’ was significantly higher compared to 0 h in both arterial (p = 0.004) and portal (p = 0.010) blood, but there was no significant difference in endotoxin concentration between portal and arterial blood at any time point. Endotoxin concentrations in APAP pigs were 2.6 ± 1.6 endotoxin units (EU)/ml and 14.3 ± 1.6 EU/ml in portal blood at 0 h and ‘ALF’ respectively and 2.9 ± 1.5 EU/ml and 13.4 ± 1.5 EU/ml in arterial blood at 0 h and ‘ALF’ respectively. In Controls plasma endotoxin concentration was unaffected by time and independent of blood vessel sampled. Plasma endotoxin concentrations in Control pigs were 4.3 ± 2.0 EU/ml and 8.2 ± 2.0 EU/ml in portal blood at 0 h and 20 h respectively and 5.5 ± 2.1 EU/ml and 6.8 ± 2.1 EU/ml in arterial blood at 0 h and 20 h respectively.

### Portal vein flow is unaffected by ALI

Portal vein flow (PVF) in APAP and Control groups did not change significantly with time and no significant difference was seen between these groups. PVF in Controls was 690 ± 246 ml/min and 722 ± 107 ml/min at 4 h and 20 h respectively. PVF in APAP pigs was 896 ± 144 ml/min and 607 ± 120 ml/min at 4 h and ‘ALF’ respectively.

### Platelet count, ADAMTS-13, endotoxin, fibrinogen and PC are independently associated with TEG in ALI

There were significant strong correlations between the activities/concentrations of all of the procoagulants and anticoagulants measured, platelet count, neutrophil count and endotoxin concentration. Platelet count, as expected, had a significant independent effect on α (estimate = 0.05 ± 0.02, p = 0.006), MA (estimate = 0.03 ± 0.01, p = 0.022) and K (estimate = −0.02 ± 0.01, p = 0.039) with a fall in platelet count being associated with trend to hypocoagulability. ADAMTS-13 activity had a significant independent effect on α (estimate = −0.50 ± 0.17, p = 0.006) and K (estimate = 0.44 ± 0.17, p < 0.001) with a fall in ADAMTS-13 activity being associated with increased speed of clot formation, in agreement with the hypercoagulability and fall in ADAMTS-13 activity seen at 12 h. Endotoxin concentration had a significant independent effect on MA (estimate = −1.21 ± 0.50, p = 0.024) and R (estimate = 3.19 ± 1.13, p = 0.008), emphasising the role of endotoxin in progression of hypocoagulability. Finally, fibrinogen (estimate = 12.10 ± 3.23, p = 0.001) and protein C (estimate = 0.26 ± 0.08, p = 0.004) were independently associated with MA, emphasising the role of both procoagulants and anticoagulants in determination of coagulation status.

## Discussion

We describe for the first time the evolution of coagulation disturbance from health through ALI to established ALF, prior to the onset of multi-organ failure, in a pig model of APAP-induced ALF. TEG analysis clearly demonstrated early hypercoagulability at 12 h, progressing to hypocoagulability. In this study, a key independent event implicated in early hypercoagulability was a reduction in ADAMTS-13, which preceded the fall in the pro-coagulants (FV, FVIII) synthesised by the liver and occurred in the face of normal vWF Ag concentrations. There is disproportionate decline in platelet count suggesting potential consumption and this is associated with presence of platelet aggregates in small intrahepatic portal vessels at 12 h. Notably, portal blood flow was unchanged and there was no evidence of enhanced fibrin breakdown, distinguishing the hypercoagulability of ALF from the syndromes of reduced blood flow and disseminated intravascular coagulation (DIC) respectively. The progression of hypercoagulability to hypocoagulability was demonstrated first by changes in TEG and later by changes in thrombin generation, PT, PTT and INR. Key independent events associated with hypocoagulability in this study were increase in endotoxin and decline in platelet count, PC and fibrinogen, demonstrating again the importance of concurrent decline in procoagulants and anticoagulants. These key events were also correlated with neutrophilia with left shift and presence of a heparin-like effect, which may result from endothelial injury due to primary APAP-related liver injury, response to systemic inflammation or local hypoxic response due to platelet aggregates. This study highlights ADAMTS-13 reduction, endotoxemia and platelet aggregates in the liver as key events in the evolution of the coagulopathy in ALI.

In this study we show for the first time an *in vivo* association between hypercoagulability and an increased vWF antigen to ADAMTS13 ratio, platelet aggregates in intrahepatic portal vessels and decline in natural anticoagulants. This occurred at the 12 h time point. At this time, the greater number of intrahepatic portal platelet aggregates in the APAP pigs compared to Controls was not associated with differences in portal flow. Inflammation and endotoxemia may have been contributory factors, as they are known to activate coagulation^13–16^. However, in this study increases in neutrophil count and endotoxin concentration were not yet apparent at 12 h. Endothelial changes secondary to ALI may also have contributed to platelet aggregation^17, 18^. ADAMTS-13 is synthesised primarily by hepatic stellate cells and cleaves hyperactive multimeric or ultra-long forms of vWF into less active vWF fragments. This reduction in vWF activity appears to be greater in the presence of FVIII and platelet glycoprotein 1bα. It has previously been reported that ADAMTS-13 activity is reduced in ALF/ALI patients and plasma taken from these patients resulted in increased aggregation of normal platelets *ex vivo* compared to plasma from normal controls^22^. We report now *in vivo* evidence of this observation, which was previously made *ex vivo*. This study does not allow us to determine whether platelet microthrombi were present in other organs apart from the liver, as no other tissues were biopsied at 12 h. However, a previous clinical study, which reviewed peroperative deaths, identified pulmonary platelet aggregates as a finding particular to liver transplant surgeries, despite the absence of other risk factors for thrombosis^23^. We now report a possible mechanism for this post-mortem finding.

The importance of other anticoagulants in ALF, including PC, has also been reported previously. PC is synthesized by the liver and secreted into the circulation. It is localised to endothelial surfaces by the endothelial PC receptor. PC is activated by endothelium bound thrombomodulin (TM)-thrombin complexes and binds PS to inactivate activated FV and FVIII^24^. Reduction in hepatic PC synthesis combined with PC consumption and/or endothelial injury therefore results in a prothrombotic state. In ALF, Yamaguchi *et al*. (2006) showed increased plasma thrombin-AT complexes and free TM, decreased activated PC-PC inhibitor complexes and perhaps most importantly decreased ratio of activated PC-PC inhibitor complexes to PC^25^. They concluded that in ALF, endothelial cell injury results in decreased PC activation and a prothrombotic state. Warkentin and Pai also suggest a central role for PC deficiency in acute hypoxic liver injury, which may predispose to microthrombi and ischaemic limb necrosis^26^. Recombinant human PC is available as a therapeutic agent^24^. However, it is unlicensed in patients with liver failure due to potential risk of bleeding. Moreover, its major reported therapeutic effects appear not to be due to its anti-coagulant activity, but rather its anti-inflammatory, anti-apoptotic and endothelial stabilisation effect^24^. Therefore, consideration for use in early ALF/ALI with appropriate monitoring of coagulation status may be beneficial in selected cases^27, 28^.

Endotoxin is known to activate the coagulation cascade via a pathway which is dependent on increased tissue factor expression on circulating monocytes and release of tissue factor positive microparticles into the circulation. Experimental treatment with endotoxin results in hypercoagulability followed by hypocoagulability in man, rodents and pigs^14, 29, 30^. However contrary to our findings, the hypocoagulability of experimental endotoxemia is a consumptive coagulopathy, associated with elevation in d-dimers and DIC^14, 29, 30^.

In the current study a heparin-like effect (HLE) was observed, supporting a role for endothelial injury in the coagulopathy of ALI. We previously defined a heparin-like effect in nine out of 10 human ALF patients using TEG analysis, as a correction of R plus K of more than 50% in the presence of heparinase^18^. Anti-Xa assays can also be used to measure the effect of endogenous and exogenous heparins in plasma^31^ and the results of the chromogenic anti-Xa assay used in this study has previously been shown to be correlated with the R TEG parameter when using heparinase cups in infected cirrhotic patients^17^. In the current study, anti-Xa activity was only detected in 3 samples at time 0 h: this may have been due to contamination with unfractionated heparin sodium used to flush catheters prior to insertion. Reason for failure to detect anti-Xa activity when INR exceeded 3 in presence of significant HLE is unclear and warrants further investigation of the assay used with porcine samples.

The pig model of APAP-induced ALF used in this study has been previously shown to mimic the clinical time course of hyperacute liver failure in man^32^. In this study we confirm hepatocyte necrosis at the time INR exceeded 3, defined as ‘ALF’, by demonstrating significant elevations in HMGB1, a biomarker of hepatocyte necrosis in APAP-induced ALF in man^21^. Studies using biomarkers to map the time course of liver injury to clinical liver failure in man and pigs are yet to be published despite considerable progress in this field^21, 33^.

In conclusion, the results of this longitudinal study shows for the first time that APAP related ALI is associated with the initial development of hypercoagulability, consequent upon reduction in levels of ADAMTS-13 and natural anticoagulants, which may contribute to intrahepatic portal venous platelet aggregation and is a potential therapeutic target. Hypercoagulability progresses to hypocoagulability due to a combination of reduction in platelets, reduction in circulating procoagulants and anticoagulants and endotoxemia. The results of this study highlight reduction in ADAMTS-13, platelet aggregation and decline and endotoxemia as key events in the evolution of the coagulopathy of ALI and emphasises the importance of global assessments of coagulation status in ALI/ALF and the use of blood products on an “on-demand” basis.

## Methods

### Porcine model of acute liver failure

A previously described pig model of APAP-induced ALF was used in this study^32^. Ten ALF pigs (‘APAP pigs’) and three control pigs (‘Controls’) from a previously published study^34^, from which appropriate blood samples had been taken, were included in this study. All animal procedures were approved by the Animal Welfare and Ethical Review Board of the Royal Veterinary College, University of London and were carried out in accordance with the Animals (Scientific Procedures) Act 1986.

Female, 26–36 kg, Landrace cross Large White pigs were induced to and maintained under general anaesthesia until study end. At the beginning of the study all pigs were instrumented for repeat blood sampling including a 5Fr, 20 cm catheter in a femoral artery, a 7.5Fr, 16 cm catheter in the right external jugular vein and a 5Fr, 20 cm catheter in the portal vein via an ileal vein.

Time 0 h was defined as the time immediately following instrumentation of the pigs for experimental procedures and was just prior to onset of induction to ALF. Acute liver failure was induced in ‘APAP pigs’ with an aqueous APAP suspension, given via an oroduodenal tube as described previously^32^. A loading dose of 0.25 g/kg APAP was followed by an hourly maintenance APAP dose, adjusted between 0.5 and 4 g to achieve toxic serum APAP concentrations of greater than 300 mg/L. Progression of ALI to ALF was monitored by measuring prothrombin time (PT), initially every 4 h and then every 1 h when prolongation of PT was observed. PT was measured using venous sodium citrate whole blood samples and a bedside analyser, CoagDx (Idexx Laboratories Ltd, West Yorkshire, UK): this PT will hereafter be referred to as PT_CoagDx_. ‘ALF’ was defined as the time point at which PT_CoagDx_ exceeded 3 times that at time 0 h, which was equivalent to an INR of 3. This was based upon previous studies, where INR of 3 was associated with subsequent progression to death in 100% of pigs^32^. ‘Control pigs’ were managed using the same protocols as APAP pigs, except that they received water without APAP for the 20 h required for ‘ALF’ induction in APAP pigs.

All pigs included in these studies were ultimately sacrificed or died within 20 h of ‘ALF’ for APAP pigs and at 40 h for Control pigs. Histological examination of paraffin embedded post-mortem liver specimens was used to confirm acute APAP-induced hepatocyte necrosis in APAP pigs.

In the following studies progression of coagulopathy was assessed between Time 0 h and ‘ALF’ unless otherwise stated. During this time all pigs received intensive supportive care according to defined protocols^32^, which did not include treatments for coagulopathy nor any blood products.

### Haematology

Femoral artery blood samples were collected in Ethylenediaminetetraacetic acid (EDTA) tubes (Becton Dickinson UK Ltd, Plymouth, UK) and fresh blood smears were made for complete blood cell counts at a commercial laboratory (Pathology and Diagnostic Laboratories, RVC, UK) at 0 h, 4 h, 8 h, 12 h and then every 2 h until ‘ALF’. EDTA blood was stored at 4 °C prior to analysis within 24 h.

### Thromboelastography (TEG)

TEG was performed on the TEG analyser 5000 (Haemonetics Ltd, Coventry, UK). Baseline TEG was performed after intramuscular premedication with atropine, azaperone, midazolam and ketamine, from a blood sample obtained directly from an ear vein to achieve normal TEG values for this study. Subsequent blood samples were obtained from femoral artery and portal vein catheters at 0 h, 12 h, then every 2 h until ‘ALF’. One ml of native whole blood collected in a clean 2.5 ml plastic syringe was analysed within 4 minutes of collection. TEG analysis was carried out with (heparinase cup) and without (natural cup) addition of heparinase simultaneously. Two TEG analysers (Analyzer 5000) permitted simultaneous analysis of portal vein and arterial samples. All TEG traces were reviewed graphically and those with errors due to technical problems, e.g. bubbles in the sample, calibration error, discontinuation of analysis prior to achievement of all data points, were discarded. The following machine generated parameters were analysed: R, reaction time, representing time to initial fibrin formation; K, representing time from beginning of clot formation until thromboelastogram amplitude reaches 20 mm; α, angle, representing rapidity of fibrin build up and cross-linking; and MA, maximum amplitude, representing ultimate strength of the fibrin clot.

### Analysis of clotting times, thrombin generation, procoagulants and anticoagulants

Femoral arterial blood samples were collected at 0 h, 12 h, 16 h and ‘ALF’ from 3 Controls and 8 APAP pigs. Additionally, portal vein blood samples were collected from 2 Controls and 3 APAP pigs at the same times, except that portal vein blood was collected at 14 h instead of 16 h, due to need to limit total volume of blood collected at each time point. Blood was transferred directly to 4.5 ml, 3.2% tri-sodium citrate blood collection tubes (Becton Dickinson UK Ltd) on ice. Platelet poor plasma was prepared immediately by double centrifugation at 3500 rpm for 10 min at 4 °C. Samples were stored in 500 µl aliquots at −70 °C until analysis.

An ACL TOP Coagulometer and HaemosIL reagents from Instrumentation Laboratory (IL), Warrington, UK were used for the following assays, unless otherwise stated and as previously described^5, 35, 36^. Prothrombin time (PT_IL_), and activated partial thromboplastin time (APTT) were measured using PT-Fibrinogen HS Plus and APTT SP respectively. FV and FVIII activities were measured by one-stage PT-based and one-stage APTT-based assays respectively. Protein C (PC) and Protein S (PS) activities were measured using the chromogenic Protein C assay and the Free Protein S assay respectively. Anti-Xa activity was measured using the liquid Anti-Xa chromogenic assay. Antithrombin (AT) activity was measured using an in-house chromogenic assay.

vWF Ag was assayed using an in-house ELISA incorporating a rabbit polyclonal anti-human vWF antibody (Dako, Glostrup, Denmark) as previously described^37^. Fibrinogen was measured using the Clauss method^38^. ADAMTS-13 activity was measured using the Technozym ELISA (Technoclone, Vienna, Austria). D-dimers were measured using a pig d-dimer ELISA kit (Abbexa Ltd, Cambridge, UK).

Thrombin generation following *in vitro* activation of coagulation by 5 pmol tissue factor with 4 µM/L phospholipids was assessed using the Calibrated Automated Thrombogram method as previously reported for ‘unmodified’ thrombin generation^35^. All assay reagents were obtained from Thrombinoscope BV, Maastricht, The Netherlands and used according to their recommendations. The following automated parameters were analysed: endogenous thrombin potential (ETP, nM min), proportional to thrombin concentration and the duration of its activity; and peak height (nM), representative of total plasma thrombin-generating capacity.

### Immunohistochemistry for platelet microthrombi detection

Four-micron paraffin-embedded sections of formalin-fixed liver biopsies taken from pigs at 0 h (3 Controls and 9 APAP) and at 12 h (3 Controls and 3 APAP) were used for platelet microthrombi detection. Platelet aggregates were identified based on platelet morphology, localisation in intravascular spaces and immunohistochemical labelling using a mouse anti-human monoclonal antibody against the platelet surface antigen, CD61 (clone 2f2), obtained in a ‘ready-to-use’ format (Leica Microsystems, Cambridge, UK). The BondMax Automated System and the Bond Polymer Refine Detection System from Leica Microsystems was used according to their recommendations. Heat induced epitope retrieval was performed using Bond Epitope Retrieval Solution 1 (pH 6) for 20 minutes at 90 °C. Stained sections were reviewed blind at ×10 magnification by two researchers (KL and SP) reaching a consensus for all counts. Ten fields centred on a portal area were reviewed per section. Mean number of platelet aggregates within portal veins per field was recorded for each section. Sections were then ranked according to abundance of platelet aggregates within sinusoids and assigned a grade of 0 to 3 accordingly (0 = no sinusoidal platelet aggregates; 3 = abundant sinusoidal platelet aggregates).

### Endotoxin assays

Femoral artery and portal vein heparin plasma samples were collected from 3 Controls and 9 APAP pigs at 0 h, 8 h, 16 h and ‘ALF’ and stored at −70 °C pending analysis. Plasma endotoxin concentrations were measured using the Kinetic Turbidimetric Limulus Amebocyte Lysate Assay (Charles River Laboratories International Inc., MA, USA) according to the manufacturer’s instructions and as described previously^39^.

### High-mobility group box-1 protein, HMGB1, assays

Total HMGB1 in arterial plasma samples collected from 3 Controls and 10 APAP pigs at 0 h and ‘ALF’ and stored at −70 °C pending analysis, was quantified by enzyme-linked immunosorbent assay (ELISA, Shino-Test Corporation, Tokyo, Japan) as previously described^40^.

### Portal vein flow

Portal vein flow was recorded every 15 minutes for all pigs using a Transonic flow meter (T402-PP) and 12 mm flow probe (12PSB521) placed around the portal vein (Transonic Systems Inc., Ithaca, New York), from 4 h to ‘ALF’.

### Statistical analysis

IBM SPSS Statistics V22 (IBM Corporation, New York, USA) was used for all data analysis and significance was set at the 5% level. The Linear Mixed Models procedure was employed to analyse each recorded parameter in turn and to consider the effect of time, group (APAP or Control) and blood vessel (artery or portal vein) on each parameter. The ‘ALF’ time point for the APAP group was compared to the 20 h time point for the Control group. The Linear Mixed Models procedure was used to determine which of these parameters may act as independent determinants of the four TEG parameters recorded (α, K, R, MA) for native whole blood without heparinase. Parameters considered for inclusion in this model were platelet count, neutrophil count, endotoxin concentrations and all procoagulants and anticoagulants assayed. A backward elimination method was used for parameter selection.

### Data Availability

The datasets generated during and/or analysed during the current study are available from the corresponding author on reasonable request.

## References

[1] O’Grady JG, Alexander GJ, Hayllar KM, Williams R (1989). Early indicators of prognosis in fulminant hepatic failure. Gastroenterology.

[2] McPhail MJ, Wendon JA, Bernal W (2010). Meta-analysis of performance of Kings’s College Hospital Criteria in prediction of outcome in non-paracetamol-induced acute liver failure. J. Hepatol..

[3] Vaquero J (2005). Complications and use of intracranial pressure monitoring in patients with acute liver failure and severe encephalopathy. Liver Transpl..

[4] Munoz SJ, Stravitz RT, Gabriel DA (2009). Coagulopathy of acute liver failure. Clin. Liver Dis..

[5] Agarwal B (2012). Evaluation of coagulation abnormalities in acute liver failure. J. Hepatol..

[6] Stravitz RT (2012). Minimal effects of acute liver injury/acute liver failure on hemostasis as assessed by thromboelastography. J. Hepatol..

[7] Tripodi A, Mannucci PM (2011). The coagulopathy of chronic liver disease. N. Engl. J. Med..

[8] Lisman T, Stravitz RT (2015). Rebalanced Hemostasis in Patients with Acute Liver Failure. Semin. Thromb. Hemost..

[9] Lisman T (2012). Intact thrombin generation and decreased fibrinolytic capacity in patients with acute liver injury or acute liver failure. J. Thromb. Haemost..

[10] Stravitz RT (2013). Role of procoagulant microparticles in mediating complications and outcome of acute liver injury/acute liver failure. Hepatology.

[11] Habib M (2014). Evidence of rebalanced coagulation in acute liver injury and acute liver failure as measured by thrombin generation. Liver Int..

[12] Agarwal B, Shaw S, Shankar Hari M, Burroughs AK, Davenport A (2009). Continuous renal replacement therapy (CRRT) in patients with liver disease: is circuit life different?. J. Hepatol..

[13] van Deventer SJ (1990). Experimental endotoxemia in humans: analysis of cytokine release and coagulation, fibrinolytic, and complement pathways. Blood.

[14] Mayr FB, Jilma B (2006). Coagulation interventions in experimental human endotoxemia. Transl. Res..

[15] Spiel AO, Mayr FB, Firbas C, Quehenberger P, Jilma B (2006). Validation of rotation thrombelastography in a model of systemic activation of fibrinolysis and coagulation in humans. J. Thromb. Haemost..

[16] Ostrowski SR (2013). Discrepant fibrinolytic response in plasma and whole blood during experimental endotoxemia in healthy volunteers. PLoS One.

[17] Zambruni A (2004). Endogenous heparin-like activity detected by anti-Xa assay in infected cirrhotic and non-cirrhotic patients. Scand. J. Gastroenterol..

[18] Senzolo M (2009). Heparin-like effect contributes to the coagulopathy in patients with acute liver failure undergoing liver transplantation. Liver Int..

[19] Agarwal B (2013). Hemostasis in patients with acute kidney injury secondary to acute liver failure. Kidney Int..

[20] von Kanel R, Dimsdale JE (2000). Effects of sympathetic activation by adrenergic infusions on hemostasis *in vivo*. Eur. J. Haematol..

[21] Antoine DJ (2013). Mechanistic biomarkers provide early and sensitive detection of acetaminophen-induced acute liver injury at first presentation to hospital. Hepatology.

[22] Hugenholtz GC (2013). An unbalance between von Willebrand factor and ADAMTS13 in acute liver failure: implications for hemostasis and clinical outcome. Hepatology.

[23] Sankey EA (1993). Pulmonary platelet aggregates: possible cause of sudden peroperative death in adults undergoing liver transplantation. J. Clin. Pathol..

[24] Griffin JH, Zlokovic BV, Mosnier LO (2015). Activated protein C: biased for translation. Blood..

[25] Yamaguchi M (2006). Decreased protein C activation in patients with fulminant hepatic failure. Scand. J. Gastroenterol..

[26] Warkentin TE, Pai M (2016). Shock, acute disseminated intravascular coagulation, and microvascular thrombosis: is ‘shock liver’ the unrecognized provocateur of ischemic limb necrosis?. J. Thromb. Haemost..

[27] Rinaldi L (2008). Use of activated protein C in liver transplantation patients with septic shock. Liver Transpl..

[28] Rewari V, Milan ZB, Attia M, Davies M (2011). Recombinant human activated protein C in a liver transplant recipient in the immediate postoperative period. Anaesth. Intensive Care.

[29] Schochl H (2011). Thromboelastometry (TEM) findings in disseminated intravascular coagulation in a pig model of endotoxinemia. Mol. Med..

[30] Tsai HJ (2012). Application of thrombelastography in liver injury induced by endotoxin in rat. Blood Coagul. Fibrinolysis..

[31] Gehrie E, Laposata M (2012). Test of the month: The chromogenic antifactor Xa assay. Am. J. Hematol..

[32] Lee KC (2013). A reproducible, clinically relevant, intensively managed, pig model of acute liver failure for testing of therapies aimed to prolong survival. Liver Int..

[33] Baker LA (2015). Circulating microRNAs Reveal Time Course of Organ Injury in a Porcine Model of Acetaminophen-Induced Acute Liver Failure. PLoS One.

[34] Lee KC (2015). Extracorporeal liver assist device to exchange albumin and remove endotoxin in acute liver failure: Results of a pivotal pre-clinical study. J. Hepatol..

[35] Gatt A (2010). Enhanced thrombin generation in patients with cirrhosis-induced coagulopathy. J. Thromb. Haemost..

[36] Chowdary P (2015). Thrombin generation assay identifies individual variability in responses to low molecular weight heparin in pregnancy: implications for anticoagulant monitoring. Br. J. Haematol..

[37] Riddell AF (2002). Use of the collagen-binding assay for von Willebrand factor in the analysis of type 2M von Willebrand disease: a comparison with the ristocetin cofactor assay. Br. J. Haematol..

[38] Clauss A (1957). [Rapid physiological coagulation method in determination of fibrinogen]. Acta Haematol..

[39] Jalan R (2011). Acute endotoxemia following transjugular intrahepatic stent-shunt insertion is associated with systemic and cerebral vasodilatation with increased whole body nitric oxide production in critically ill cirrhotic patients. J. Hepatol..

[40] Antoine DJ (2009). High-mobility group box-1 protein and keratin-18, circulating serum proteins informative of acetaminophen-induced necrosis and apoptosis *in vivo*. Toxicol. Sci..

